# A Self-Powered Flexible Displacement Sensor Based on Triboelectric Effect for Linear Feed System

**DOI:** 10.3390/nano13243100

**Published:** 2023-12-07

**Authors:** Tingting Zhao, Dongsheng Li, Peijuan Cui, Zhongbin Zhang, Yuyang Sun, Xingyou Meng, Zhanlin Hou, Zaiping Zheng, Yuping Huang, Huicong Liu

**Affiliations:** 1 Jiangsu Provincial Key Laboratory of Advanced Robotics, Robotics and Microsystems Center, School of Mechanical and Electrical Engineering, Soochow University, Suzhou 215123, China; 20214229013@stu.suda.edu.cn (T.Z.); dsli@suda.edu.cn (D.L.);; 2 Laboratory of Aerospace Servo Actuation and Transmission, Beijing Institute of Precision Mechatronics and Controls, Beijing 100076, China

**Keywords:** flexible displacement sensor, self-powered, linear feed system, triboelectric effect

## Abstract

The detection and feedback of displacement and velocity significantly impact the control accuracy of the linear feed system. In this study, we propose a flexible and self-powered displacement sensor based on the triboelectric effect, designed for seamless integration into linear feed systems. The displacement sensor comprises two parts, the mover and stator, operating in a sliding mode. This sensor can precisely detect the displacement of the linear feed system with a large detection range. Additionally, the sensor is capable of real-time velocity detection of linear feed systems, with an error rate below 0.5%. It also offers advantages, such as excellent flexibility, compact size, stability, easy fabrication, and seamless integration, with linear feed systems. These results highlight the potential of the self-powered displacement sensor for various applications in linear feed systems.

## 1. Introduction

Linear feed systems play a pivotal role in diverse fields, encompassing intelligent manufacturing, aerospace, and medical equipment [1,2,3,4]. The precision of machine tool machining and the accuracy of servo actuator control are significantly influenced by the detection and feedback of displacement and velocity for typical linear feed systems, such as linear motors and ball screws [5,6,7]. Consequently, it becomes imperative to incorporate a displacement sensor with high accuracy and a large detection range. Additionally, adherence to other requisites, such as compact size, ease of integration, robust stability, and cost-effectiveness, should be tailored to specific application scenarios [8,9,10,11,12,13].

At present, the displacement sensors employed in linear feed systems primarily include magnetic grating displacement sensors, laser displacement sensors, and grating displacement sensors [14,15,16]. Magnetic grating displacement sensors offer advantages, such as low cost and ease of operation, but they are susceptible to demagnetization due to external magnetic field interference, resulting in relatively poor stability [14]. Laser displacement sensors have gained widespread attention due to their high accuracy, rapid response, and capability to perform various detections, including displacement, velocity, and angular velocity [15]. Nevertheless, the large volume of their transmitters hinders direct integration into narrow spaces. Grating displacement sensors offer a substantial detection range and high accuracy but come with a higher cost, low tolerance to vibration, and the need for precise installation [16]. Despite the widespread use of these displacement sensors, their relatively large size renders them unsuitable for integration into narrow spaces and curved surfaces.

In recent years, flexible sensors have garnered significant attention due to their compact size, flexibility, and applicability to curved surfaces [17,18,19,20,21]. The triboelectric effect, rooted in the coupling of contact charging and electrostatic induction, has demonstrated substantial potential in the realm of flexible displacement detection. For instance, Zhu et al. proposed an angular displacement sensor utilizing the triboelectric effect to monitor the bending motion of human joints with low power consumption [22]. Zhou et al. showcased a self-powered, one-dimensional displacement and speed-sensing technology based on the coupling of the triboelectric effect and electrostatic induction, achieving high resolution, a broad dynamic range, and extensive detection capabilities [23]. Li et al. designed an innovative self-powered angle/displacement sensor, leveraging the coupling effect between triboelectrification and electrostatic induction. This sensor not only detects angle/displacement but also distinguishes the real-time direction of the freestanding electrode displacement [24]. These studies offer valuable insights for the displacement detection of linear feed systems.

**Figure 1:**
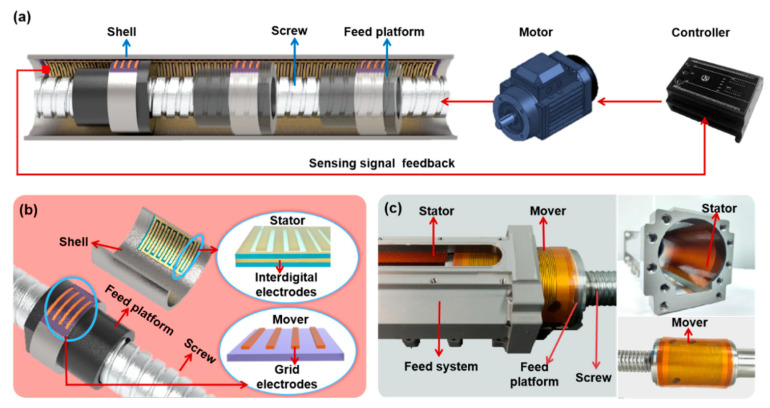
(**a**) Diagram of the displacement sensor integrated in a linear feed system; (**b**) structure diagram of the displacement sensor; (**c**) a photograph of the sensor integrated in a linear feed system.

In this study, we developed a flexible and self-powered displacement sensor based on the triboelectric effect. This sensor is designed for real-time displacement and velocity detection in linear feed systems, offering ease of fabrication and integration into narrow spaces. In a typical linear feed system, as illustrated in Figure 1a, the controller is responsible for managing the motor’s operation, thereby driving the rotation of the screw and the linear feed of the nut. Our proposed flexible displacement sensor can be seamlessly installed within the confined space of the linear feed system, enabling efficient detection of displacement and velocity. The controller receives feedback signals from the flexible displacement sensor to regulate the motor’s operation in a closed-loop system. As depicted in Figure 1b, the flexible displacement sensor comprises a mover attached to the moving feed platform and a stator fixed to the shell. The mover consists of grid electrodes on a PI film, while the stator incorporates interdigital electrodes (IDEs) sandwiched between two PI layers. The electrode of the mover is affixed to the surface of the stator, and the top PI film of the stator, in contact with the mover electrode, acts as the triboelectric layer. Operating in a sliding mode, the displacement sensor, based on the triboelectric effect, generates an electrical signal when the feed platform moves forth and back. This signal is then fed back to the controller. Figure 1c provides a photograph of the sensor seamlessly integrated into a linear feed system. The stator is attached to the shell, while the mover is affixed to the feed platform. This integration enables the real-time detection of displacement and velocity for the feed nut at different feed velocities.

## 2. Design and Fabrication

### 2.1. Working Principle of the Displacement Sensor

**Figure 2:**
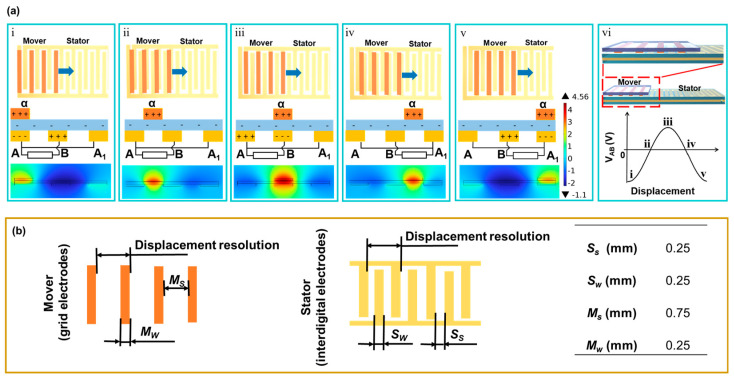
Working principle and structure of the displacement sensor: (**a**) working principle of the displacement sensor; (**b**) electrode structures of the mover and stator.

Figure 2a illustrates the operational diagram of the displacement sensor and the process of generating the triboelectric output signal (voltage difference between the stator electrodes, *V_AB_*). The displacement sensor comprises two components, the mover and the stator, and its working principle is associated with contact electrification and in-plane sliding-induced charge transfer [23,25,26]. When the mover’s electrode makes contact with the surface of the stator and slides from left to right, the triboelectric output signal is generated. Due to the difference in triboelectric polarities, as the mover starts to slide, its electrode becomes positively charged, while the PI film becomes negatively charged. When the electrodes of the mover (α) align with electrode A of the IDE in the stator (step i), there is no charge transfer between electrodes A and B due to electrostatic balance [23]. As the mover continues to slide to step ii, the original electrostatic equilibrium is disrupted, leading to electrons flowing from electrode A to B, establishing a new equilibrium state. As the mover further slides to step iii, electrons continue to flow from electrode A to B, resulting in a continuous increase in *V_AB_* [27]. This completes half of the cycle for generating the triboelectric output signal. Similarly, as the mover continues to slide to step v, electrons flow back from electrode B to electrode A_1_ until the mover’s electrode (α) coincides with electrode A_1_ of the stator. The mover returns to its original state, completing a full cycle of output signal generation. Figure 2a(vi) illustrates the corresponding output signal (*V_AB_*) of the triboelectric sensor during a sliding period.

Resolution is the minimum detectable displacement, which is determined by the period distance of the sensor electrode. Figure 2b shows the electrode width and spacing of the mover and stator (*M_w_*, *M_s_*, *S_w_*, and *S_s_*), respectively. The resolution of the displacement sensor designed in this work is 1 mm, and *M_w_* = 0.25 mm, *M_s_* = 0.75 mm, and *S_s_* = *S_w_* = 0.25 mm. The periods for electrodes of the mover and stator are consistent, that is, 2*S_s_* + 2*S_w_* = *M_s_* + *M_w_*. This ensures a well-matched configuration between the movers and stators.

### 2.2. Fabrication Process of the Displacement Sensor

**Figure 3:**
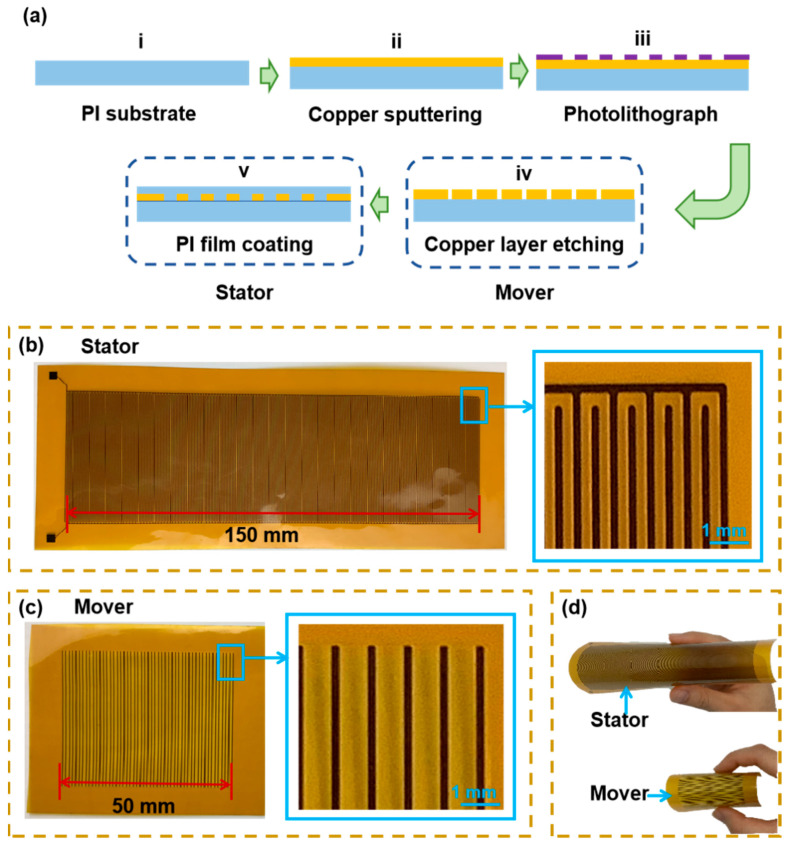
(**a**) Fabrication of the displacement sensor; (**b**) optical image of the stator; (**c**) optical image of the mover; (**d**) the flexibility of the displacement sensor.

Figure 3a depicts the fabrication process of the displacement sensor. Copper was chosen as the electrode material, and PI was selected as the triboelectric material for its excellent abrasion resistance [28]. The sensor’s preparation began with a PI film (i). Initially, a layer of copper was sputter-deposited onto the PI film surface (ii). Subsequently, the electrode with a grid structure was obtained through photolithography and an etching process (iii and iv), representing the preparation steps for the mover. For the stator, an electrode with an interdigital structure was prepared. Building on the first four steps, a PI insulating film was affixed to the electrode surface to shape the stator. Optical images of the fabricated stator and mover are presented in Figure 3b and Figure 3c, respectively. The mover in this design measures 50 mm in length, 50 mm in width, and 0.06 mm in thickness, with a volume of only 150 mm^3^. Similarly, the stator has dimensions of 150 mm in length, 50 mm in width, and 0.09 mm in thickness, with a volume of only 675 mm^3^. The flexibility of the displacement sensor is demonstrated in Figure 3d, revealing its potential to integrate with a curved surface in a narrow space. It is seen that even on an extremely complex and irregular surface between the nut and shell, the flexible displacement sensor shows great adaptability for integration with the linear feed system.

### 2.3. Experimental Setup

**Figure 4:**
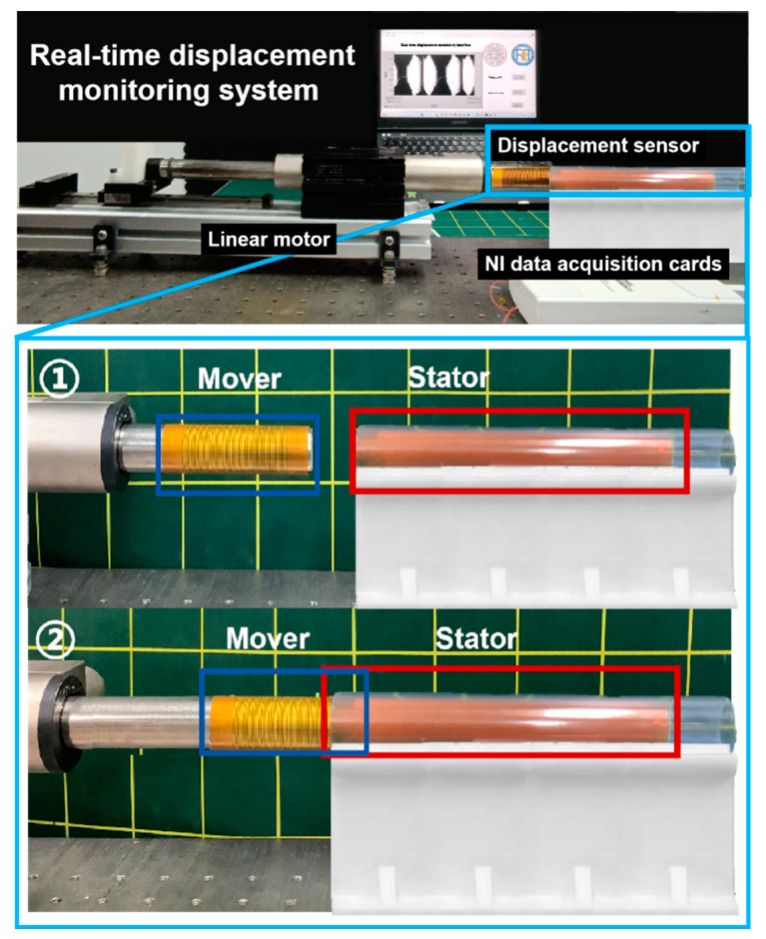
Experimental setup diagram of displacement sensor.

Figure 4 illustrates the experimental setup for real-time displacement and velocity sensing calibration. The setup primarily comprises a linear motor (LinMot K05-Y/C-4), a data acquisition card (NI USB-6210), and a LabVIEW real-time monitoring interface. The mover of the displacement sensor was affixed to the linear motor, while the stator was attached to a tube using a 3D-printed bracket. The pair of electrodes extending from the stator is non-specific polarity and is connected to the AI0 and AI8 ports of the NI data acquisition card. This configuration enables precise acquisition and monitoring of experimental data. As the linear motor operates at different velocities, the mover on the motor slides along the surface of the stator, generating a self-powered sensing signal. The NI data acquisition card collects and transmits this signal to the LabVIEW monitoring interface. Consequently, the output voltage signal of the flexible displacement sensor is displayed in real time.

## 3. Results and Discussion

**Figure 5:**
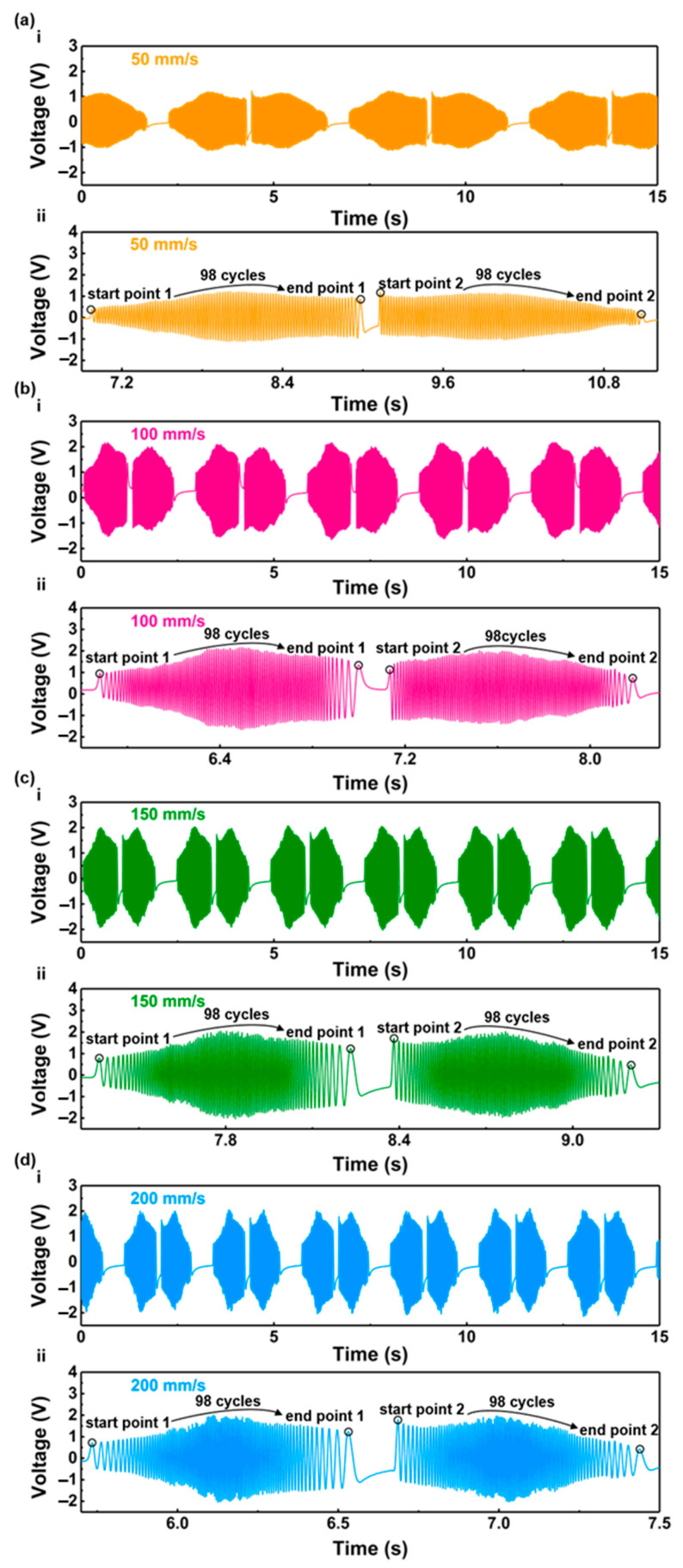
Output voltage of the displacement sensor at different velocities: (**a**) 50 mm/s; (**b**) 100 mm/s; (**c**) 150 mm/s; (**d**) 200 mm/s.

In this study, we assessed the performance of the displacement sensor in reciprocating motion. To evaluate its capabilities across various velocities, the feed velocities of the linear motors were set at 50 mm/s, 100 mm/s, 150 mm/s, and 200 mm/s, respectively. The corresponding voltage outputs of the flexible displacement sensor are depicted in Figure 5a–d. The maximum voltage outputs were recorded as 1.40 V, 2.12 V, 2.13 V, and 2.22 V at velocities of 50 mm/s, 100 mm/s, 150 mm/s, and 200 mm/s, respectively. Figure 5a(ii)–d(ii) illustrate the sensor’s output voltage during a single motion cycle at different velocities. The output voltage of the sensor increases with the increase in velocity, but the trend of the increase in the output voltage decreases when the velocity exceeds a certain value.

Based on the working principle of the designed triboelectric displacement sensor, the sensor’s resolution is defined as 1 mm, indicating that the displacement sensor generates a sinusoidal cycle signal for every 1 mm it traverses. By calculating the cycle number of sine signals, the actual displacement of the feed system can be determined. This relationship is expressed in Equation (1), where *D* represents displacement, *N* is the cycle number of sine signals, and *R* is the sensor’s resolution.

(1) D=NR

As illustrated in Figure 5a(ii)–d(ii), there are 98 sine cycles from the starting point (start point 1) to the ending point (end point 1) during the forward stroke, and 98 sine signals from the starting point (start point 2) to the ending point of the reverse stroke (end point 2). This observation implies that the reset displacement of the linear feed system is 98 mm. Despite variations in the voltage peak generated by the sensor at different velocities, the displacements detected by the sensor remain consistent, affirming that the accuracy of displacement detection is unaffected by velocity.

**Figure 6:**
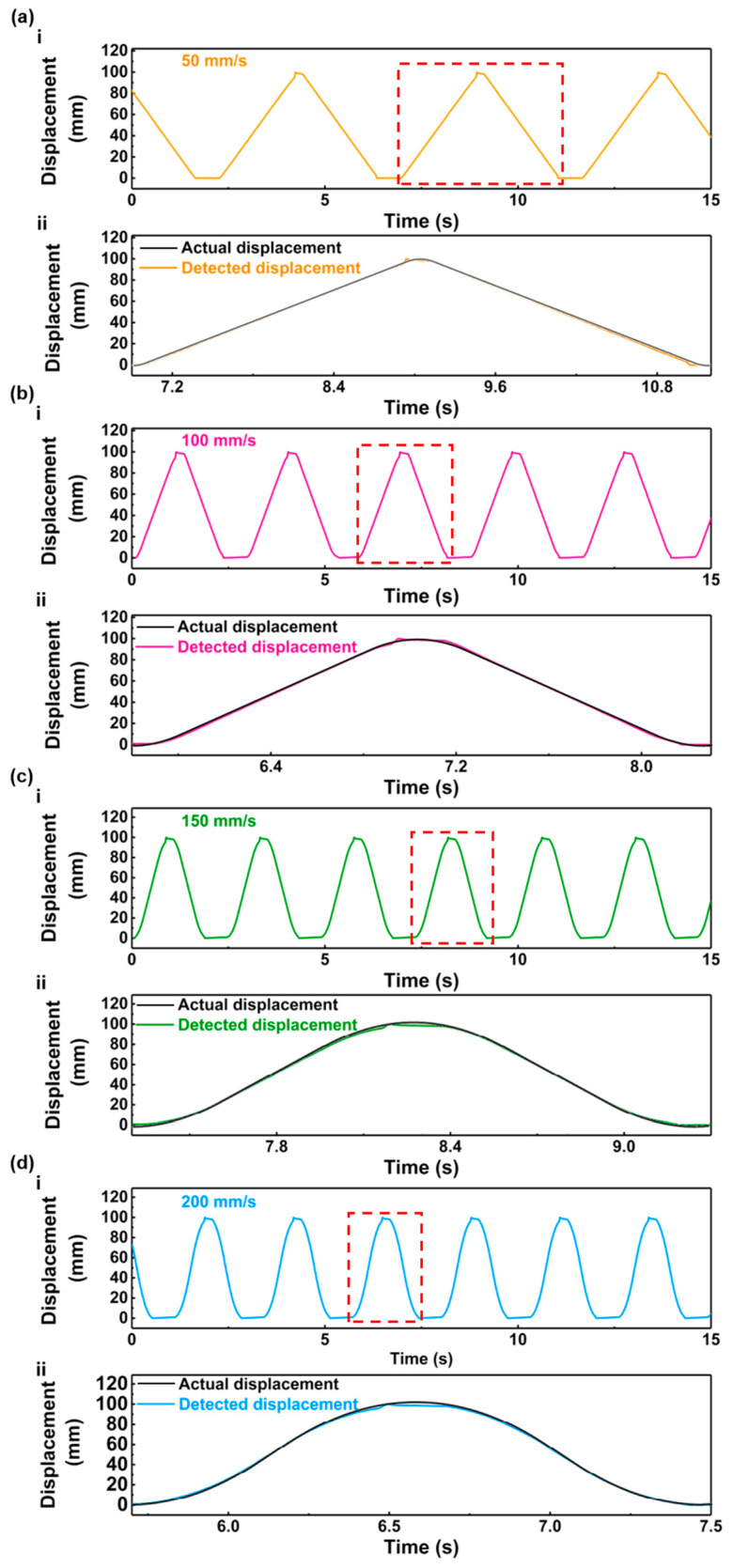
The displacements detected by the sensor and actual displacements at different velocities: (**a**) 50 mm/s; (**b**) 100 mm/s; (**c**) 150 mm/s; (**d**) 200 mm/s.

The real-time displacement of the linear feed system can be determined based on the output of the displacement sensor, as illustrated in Figure 5 and Equation (1). The displacement curves are presented in Figure 6. The detected displacements during a single reciprocating motion at different velocities are compared with the actual displacements of the linear motor, as shown in Figure 6a(ii)–d(ii). The displacements detected by the displacement sensor exhibit a high degree of consistency with the actual displacements of the linear motor, indicating the accurate detection capability of the displacement sensor.

Furthermore, velocity can also be calculated based on the output of the sensor. The velocity is the distance traveled per unit of time, i.e., *V =* Δ*S*/Δ*t*. Because the resolution of the sensor is 1 mm, the time for the linear feed system with a 1 mm feed is the duration of the signal of a sinusoid cycle. Therefore, the velocity of the linear feed system in terms of the resolution (1 mm) of the sensor is shown in Equation (2), where *V* is velocity, Δt is the duration of each sinusoid cycle of the sensor output, and *R* is the resolution of the sensor.

(2) V=R/Δt

**Figure 7:**
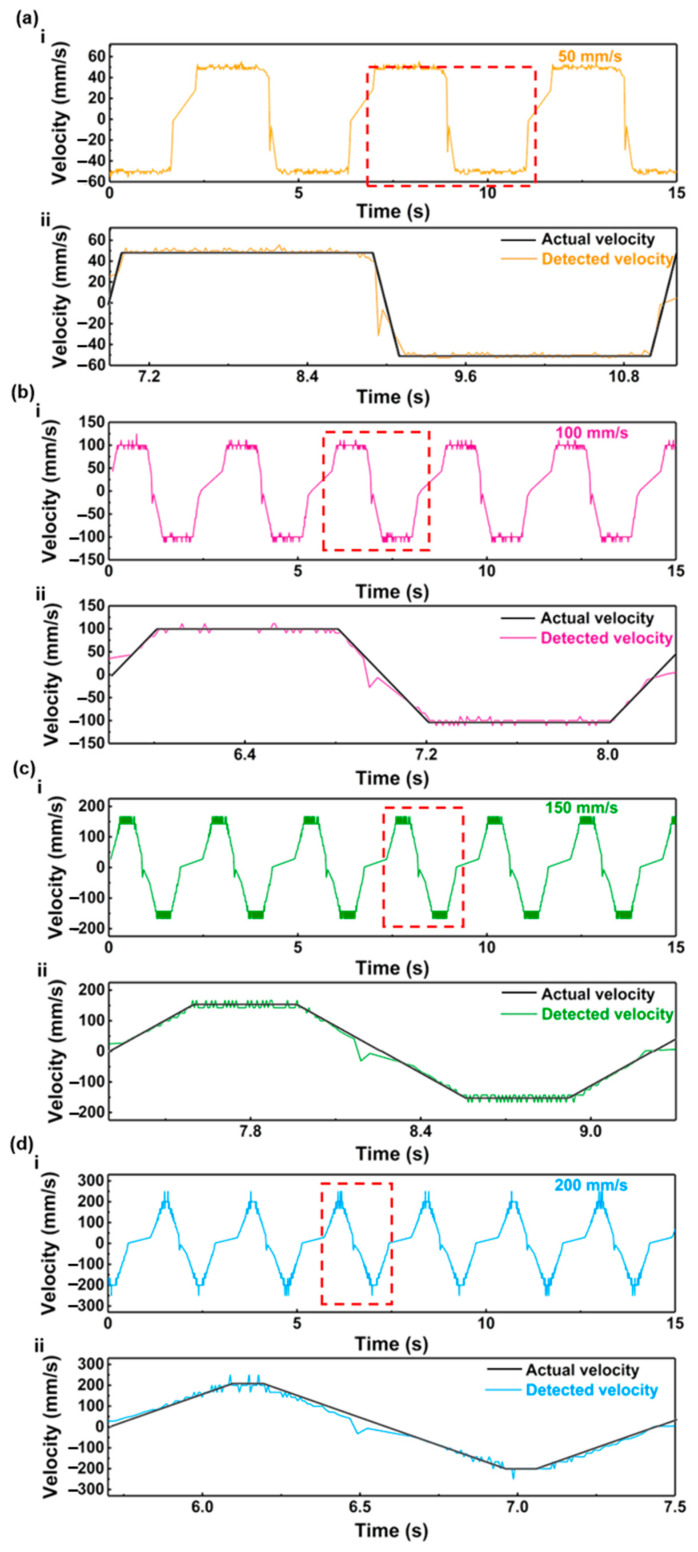
The velocities detected by the flexible sensor and actual velocities: (**a**) 50 mm/s; (**b**) 100 mm/s; (**c**) 150 mm/s; (**d**) 200 mm/s.

The real-time velocity of the sensor can be calculated based on the voltage output of the displacement sensor shown in Figure 5 and Equation (2). The real-time velocity curves are shown in Figure 7. Figure 7a(i)–d(i) illustrate the velocity detected by the displacement sensor. The detected velocity and the actual velocity in a single reciprocating motion of the linear motor are shown in Figure 7a(ii)–d(ii). The average velocities detected by the sensor during the constant velocity periods were 49.95 mm/s, 100.12 mm/s, 150.09 mm/s, and 200.79 mm/s for velocities of 50 mm/s, 100 mm/s, 150 mm/s, and 200 mm/s, respectively. Therefore, the errors of the velocity detections were 0.1%, 0.12%, 0.06%, and 0.40%, respectively. The velocities detected by the displacement sensor are consistent with the actual velocities of the linear motor, indicating that the sensor also has the capability for accurate velocity detection.

**Figure 8:**
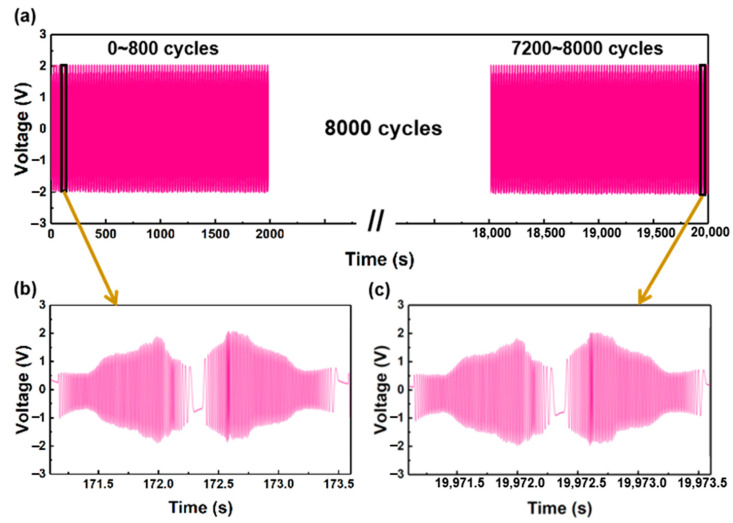
Stability test for the displacement sensor: (**a**) 0 s–20,000 s; (**b**) 70 cycles; (**c**) 7990 cycles.

Stability is a crucial parameter for the displacement sensor, ensuring the accuracy and consistency of measurement data. The stability test of the displacement sensor was conducted at a velocity of 100 mm/s for over five hours, as depicted in Figure 8a. The sensor’s output voltage remains stable after 8000 reciprocating movements, demonstrating its excellent stability. Figure 8b and Figure 8c display the output voltage during the 70th and 7990th cycles of the reciprocating motion, respectively. The sensor’s output voltages consistently align throughout the 8000 cycles of reciprocating movements. These results affirm that the displacement sensor exhibits exceptional stability for practical applications.

## 4. Conclusions

In summary, this manuscript introduces a triboelectric-based, flexible, and self-powered sensor that finds application in real-time displacement detection within linear feed systems. Comprising a mover and a stator and operating in sliding mode, the sensor produces periodic output voltage corresponding to displacement and velocity. The performance of the displacement sensor underwent a comprehensive systematic investigation, validating its precise detection capabilities for linear feed system displacements. Furthermore, the sensor demonstrates the ability for real-time velocity detection, with an accuracy exceeding 99.5%. Rigorous testing also substantiated the exceptional stability of the sensor. The sensor boasts additional advantages, including flexibility, cost-effectiveness, robust stability, compact form factor, and straightforward fabrication, thereby showcasing its potential for integration into linear feed systems across diverse fields, including intelligent manufacturing, aerospace, and medical equipment.

## Data Availability

Please contact the corresponding author for related data.
